# Biodistribution of mesenchymal stem cell-derived extracellular vesicles in a model of acute kidney injury monitored by optical imaging

**DOI:** 10.3892/ijmm.2014.1663

**Published:** 2014-02-20

**Authors:** CRISTINA GRANGE, MARTA TAPPARO, STEFANIA BRUNO, DEVASIS CHATTERJEE, PETER J. QUESENBERRY, CIRO TETTA, GIOVANNI CAMUSSI

**Affiliations:** 1 Department of Medical Sciences, University of Torino, Torino, Italy; 2 Translational Center for Regenerative Medicine, University of Torino, Torino, Italy; 3 Department of Molecular Biotechnology and Health Science, University of Torino, Torino, Italy; 4 Department of Medicine, The Warren Alpert Medical School of Brown University, Providence, RI, USA; 5 EMEA LA Medical Board, Fresenius Medical Care, Bad Homburg, Germany

**Keywords:** microvesicles, exosomes, mesenchymal stem cells, kidney

## Abstract

Mesenchymal stem cells (MSCs) contribute to the recovery of tissue injury, providing a paracrine support. Cell-derived extracellular vesicles (EVs), carrying membrane and cytoplasmatic constituents of the cell of origin, have been described as a fundamental mechanism of intercellular communication. We previously demonstrated that EVs derived from human MSCs accelerated recovery following acute kidney injury (AKI) *in vivo*. The aim of the present study was to investigate the biodistribution and the renal localization of EVs in AKI. For this purpose, two methods for EV labeling suitable for *in vivo* tracking with optical imaging (OI), were employed using near infrared (NIR) dye (DiD): i) labeled EVs were generated by MSCs pre-incubated with NIR dye and collected from cell supernatants; ii) purified EVs were directly labeled with NIR dye. EVs obtained with these two procedures were injected intravenously (i.v.) into mice with glycerol-induced AKI and into healthy mice to compare the efficacy of the two labeling methods for *in vivo* detection of EVs at the site of damage. We found that the labeled EVs accumulated specifically in the kidneys of the mice with AKI compared with the healthy controls. After 5 h, the EVs were detectable in whole body images and in dissected kidneys by OI with both types of labeling procedures. The directly labeled EVs showed a higher and brighter fluorescence compared with the labeled EVs produced by cells. The signal generated by the directly labeled EVs was maintained in time, but provided a higher background than that of the labeled EVs produced by cells. The comparison of the two methods indicated that the latter displayed a greater specificity for the injured kidney.

## Introduction

Cell-derived extracellular vesicles (EVs) contribute to intercellular communication by transferring proteins, bioactive lipids and nucleic acids (1,2). EVs include exosomes released from multivesicular bodies and microvesicles shed from the cell surface. Both vesicle types contain membrane and cytoplasmic constituents of the cells of origin (3). In particular, EVs released from stem cells have been shown to transfer, following receptor-mediated incorporation, into target cells, mRNAs and miRNAs (4–8). Several studies have indicated that the regenerative potential of stem cell-based therapy is related to paracrine/endocrine mechanisms (9–11). EVs play a critical role in transferring regenerative signals from stem cells to the injured tissues (12–14).

Acute kidney injury (AKI) involves the rapid loss of kidney function consequent to a number of causes, which represents one of the main causes of morbidity and mortality in hospitalized patients. Moreover, AKI frequently evolves into chronic renal dysfunction (15). Bone marrow-derived mesenchymal stem cells (MSCs) have been found to improve recovery following AKI induced by toxic agents and ischemia/reperfusion injury (16–18). The observation that MSC-conditioned medium mimics the effect of cell treatment in AKI has suggested a role of MSC-derived factors in coordinating the repair process (11).

Previously, we found that EVs derived from human MSCs accelerated recovery following AKI in SCID mice in a manner comparable to the cells (13,19,20). Moreover, EVs, following incorporation into renal tubular epithelial cells, have been shown to transfer specific mRNA subsets and trigger a regenerative program (13).

To evaluate whether MSC-derived EVs may represent a potential therapeutic tool for AKI, it is essential to investigate *in vivo* their biodistribution and recruitment within the injured kidneys. Optical imaging (OI) offers the potential for a non-invasive study of different targets within the body of living animals. Recently, the OI technique has been improved with the possibility of visualizing a few labeled cells *in vivo* by using new dyes (21–24). Good candidate dyes to maximize the depth of tissue penetration and reduce the background are near-infrared (NIR) fluorophores (700–900 nm); the absorption coefficient of tissue is very low and light possesses a high potential for penetration (25–27).

The aim of this study was to use OI as a technique to visualize *in vivo* the biodistribution and localization of EVs derived from MSCs in AKI within 24 h post-injection (glycerol). For this purpose, we compared two different labeling procedures, one based on direct EV labeling (DL-EV), and the other on the production of labeled EVs by donor cells pre-treated with the dye (LCD-EV).

## Materials and methods

### EV isolation

The MSCs were supplied by Lonza (Lonza, Basel, Switzerland) and cultured in the presence of MSC basal medium (MSCBM; Lonza). MSC-derived EVs were collected from the supernatant of MSCs cultured overnight in RPMI-1640 (Lonza) supplemented with 0.5% of BSA (Sigma-Aldrich, St. Louis, MO, USA). The cell supernatant was centrifuged twice at 3,000 × g for 20 min to remove cell debris and then ultracentrifuged at 100,000 × g (Beckman Coulter Optima L-90K ultracentrifuge; Beckman Coulter, Brea, CA, USA) for 1 h at 4ºC. EVs were stored in serum-free RPMI-1640 supplement with 1% DMSO at −80ºC. EV protein content was quantified by the Bradford method (Bio-Rad, Hercules, CA, USA).

### Labeling procedure

Two labeling protocols were used: i) Cells were stained in suspension and incubated with 5 μM Vybrant Cell Tracers DiD [excitation (Ex), 640 nm; emission (Em), 700 nm] or DiI (Ex, 530 nm; Em, 580 nm) (Molecular Probes, Eugene, OR, USA) solution without serum for 20 min at 37ºC. Cells were then washed in complete medium by centrifugation and cultivated for 24 h prior to supernatant collection. i) EVs were isolated by ultracentrifugation as previously described (LCD-EVs) (4,13). ii) EVs were directly labeled with 1 μM Vybrant Cell Tracers DiI or DiD during the ultracentrifugation procedure (DL-EVs) and then washed twice by ultracentrifugation in 1X phosphate-buffered saline (PBS) (28).

### EV characterization

EVs labeled with the two methods were characterized by cytofluorimetric analysis using FITC- or PE-conjugated antibody against CD44, CD105, CD90 and α5-integrin. FITC or PE mouse non-immune isotypic IgG (Miltenyi Biotec, Bergisch Gladbach, Germany) were used as the controls. Briefly, EVs (10 μg) were incubated for 15 min at 4ºC with antibodies in 100 μl and then diluted in 300 μl and immediately acquired. FACS analysis was performed using a guava easyCyte Flow Cytometer (Millipore, Billerica, MA, USA) and analyzed with InCyte software, as previously described (29,30).

The size distribution of the LCD-EVs and DL-EVs was analyzed using a NanoSight LM10 instrument (NanoSight Ltd., Amesbury, UK) equipped with the nanoparticle tracking analyses (NTA) 2.0 analytic software.

### In vitro uptake of EVs by human renal tubular epithelial cells

Human renal proximal tubular epitheial cells (PTECs) were labeled following the manufacturer’s instructions with the CFSE green dye (Vybrant CFDA SE Cell Tracer kit; Molecular Probes). Cells were incubated for 5 h at 37ºC with 50 μg/ml DL-EVs or LCD-EVs and after washing the cells were fixed in 3.5% paraformaldehyde containing 2% sucrose. Confocal microscopy analysis was performed using a Zeiss LSM 5 Pascal model confocal microscope (Carl Zeiss, Oberkochen, Germany). Hoechst 33258 dye (Sigma-Aldrich) was added for nuclear staining.

### Mouse model of AKI

Studies were conducted in accordance with the national guidelines and regulations and were approved by the Ethics Committee of the University of Torino. Male CD1 nude mice (6–8 weeks old) (Charles River Laboratories, Lyon, France), were fed for 1 week with a special diet (AIN 79; Mucedola, Settimo Milanese, Italy) to reduce tissue autofluorescence. AKI was induced, as previously described (16), by an intramuscular injection of glycerol (7 ml/kg body weight of 50% glycerol solution) into the inferior hind limbs. At 3 days post-injury, the mice were injected intravenously (i.v.) with 200 μg of DiD-labeled EVs. Sixteen nude mice with AKI were treated with LCD-EVs and were sacrificed after 5 h (n=9) and 24 h (n=7). Eleven nude mice with AKI were treated with DL-EVs and were sacrificed after 5 h (n=6) and 24 h (n=5). The same amount of LCD- and DL-EVs was i.v. injected in 12 and 6 healthy mice, respectively. The animals were sacrificed after 5 h (LCD-EV, n=6; DL-EV, n=3) and 24 h (LCD-EV, n=6; DL-EV, n=3).

### In vitro OI

*In vitro* experiments were performed using the IVIS 200 small animal imaging system (PerkinElmer, Waltham, MA, USA) using the Ex filter at 640 nm and the Em filter at 700 nm. Background fluorescence was measured and subtracted by setting up a background measurement (Ex filter, 530 nm). EV samples were placed in a non-fluorescent black container and the fluorescence intensity of increasing concentrations of LCD-EVs and DL-EVs (15, 30, 50 and 100 μg) in the same volume was evaluated. Image analysis involved the designation of regions-of-interest (ROI) as the circular area of the well containing the EV concentrations to obtain the average intensity ± standard deviation (SD), as previously described (31).

### In vivo OI

*In vivo* fluorescence imaging was performed with the same wavelength as described for *in vitro* acquisition. Identical illumination settings, such as exposure time (2 sec), binning factor (factor of 4), f/stop (set to 2) and 12 fields of view, were used for acquiring all images, and fluorescence Em was normalized to photons per second per centimeter squared per steradian (p/sec/cm^2^/sr). The color image represents the spatial distribution of fluorescence within the animal overlaid on black and white photographs of the mice, collected at the same time. Images were acquired and analyzed using Living Image 4.0 software (PerkinElmer), as previously described (32).

The mice were anesthetized with 2.5% isoflurane (Merial, Lyon, France) and images were acquired in the prone and supine position after 15 min, 5 and 24 h post-EV injection. The mice with AKI and the healthy mice treated with PBS were used as blank controls for the fluorescence signal of EVs in the AKI and healthy groups, respectively. The fluorescence signal was quantified in the kidney region and in the abdominal area, in ROI draw freehand. The relative mean fluorescence intensity of each ROI was obtained by subtracting the mean fluorescence intensity of the corresponding ROI on the blank mouse from the measured mean fluorescent intensity, as previously described (22,33). Data were expressed as the average radiance ± SD.

At the end of the experiments (5 or 24 h post-EV injection), the mice were sacrificed and dissected tissues (kidneys, spleen, liver and lungs) were imaged immediately. The mean fluorescence of each tissue sample was obtained by subtracting the fluorescence intensity of corresponding tissue from the blank mouse, as previously described (33).

### Immunofluorescence

Mice were sacrificed at 5 and 24 h and confocal microscopy analysis (Leica TSC SP5 II) was performed on frozen sections for localization of DiD-labeled EVs in the kidneys. Hoechst 33258 dye (Sigma-Aldrich) was added for nuclear staining. Images were analyzed using ImageJ software.

### Statistical analysis

The results are generally expressed as the means ± SD. Statistical analysis was performed by ANOVA with Dunnet’s multi-comparison test or the Newman*-*Keuls multi-comparison or by the Student’s t-test where appropriate. A p-value of <0.05 was considered to indicate a statistically significant difference.

## Results

### In vitro OI of labeled EVs

**Figure 1:**
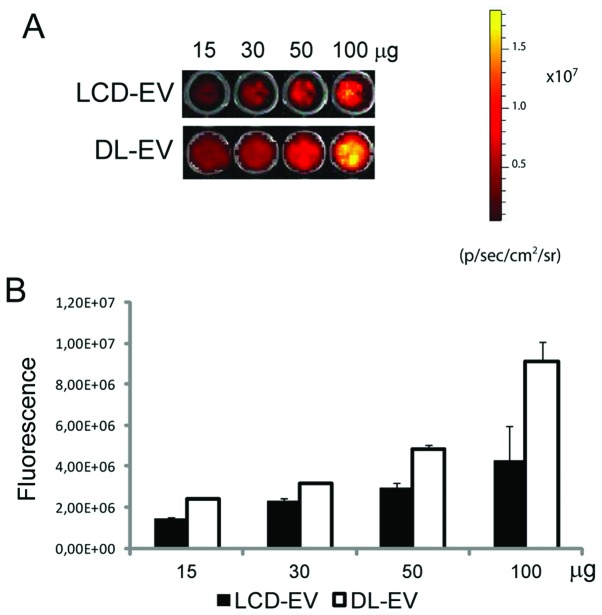
*In vitro* fluorescence assay. (A) Representative *in vitro* fluorescence images of extracellular vesicle (EV) dilutions from 15 to 100 μg of protein in 100 μl of phosphate-buffered saline (PBS). (B) Quantification of fluorescence signal calculated in the entire circle area of each well. Data are expressed as average radiance ± standard deviation (SD) of three different experiments. LCD-EV, labeled EVs produced by donor cells; DL-EV, directly labeled EVs.

The OI of EVs obtained with the two following methods was compared: i) DL-EVs were labeled with DiD after their production and purification; ii) LCD-EVs were obtained by the supernatant of MSCs previously labeled with DiD and then cultured for 24 h prior to EV collection. OI images were acquired after EV dilution, ranging from 15 to 100 μg of EV proteins, in 100 μl of PBS and the average intensity within the entire circle area of each well was calculated. The fluorescence signal correlated linearly with the EV concentration for both labeling methods. DL-EVs were brighter compared with LCD-EVs (Fig. 1).

### In vitro characterization of fluorescent EVs and incorporation into renal epithelial tubular cells

**Figure 2:**
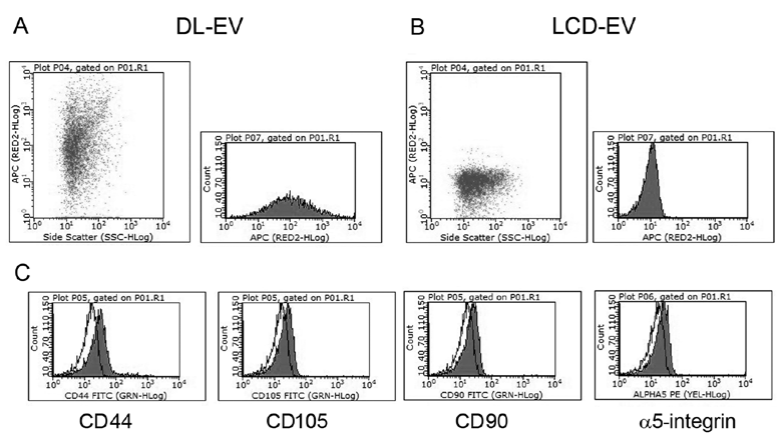
Evaluation of fluorescence of labeled cell-derived extracellular vesicles (EVs) by a Guava cytofluorimeter. (A and B). Representative dot plots and histogram plots of the fluorescent signal of labeled EVs. (A) DL-EVs show a higher fluorescence compared with (B) LCD-EVs. (C) Representative cytofluorimeter analyses of DL-EVs showing the expression of CD44, CD105, CD90 and α5-integrin. EVs produced with the two staining procedures displayed analogous expression patterns of surface markers (data not shown). White filled histograms indicate the isotypic controls. Three different EV preparations were analyzed with similar results. LCD-EV, labeled EVs produced by donor cells; DL-EV, directly labeled EVs.

**Figure 3:**
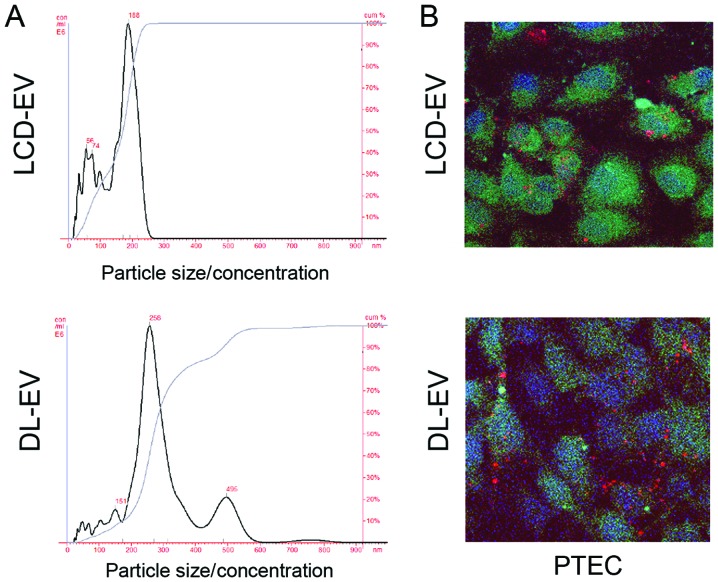
Size analysis and incorporation by renal tubular epithelial cells. (A) Representative cell-derived extracellular vesicle (EV) sizes analyzed by measurement with NanoSight. Three different preparation were analyzed with similar results. LCD-EVs maintain the same size distribution than the non-labeled EVs (180±73 nm). DL-EVs show a size of 250±89 nm with a second larger peak. (B) Representative micrographs of EV incorporation (5 h at 37ºC) in renal proximal tubular epithelial cells (PTECs). The EVs (red) produced with the two labeling procedures are equally incorporated by PTEC cells (green). Three experiments were performed with similar results. Nuclei were counterstained with DAPI (blue). Original magnification, ×630. LCD-EV, labeled EVs produced by donor cells; DL-EV, directly labeled EVs.

EVs labeled with two methods showed the same phenotype as unlabeled EVs. Cytofluorimetric analyses showed their fluorescent signal in the NIR region (Fig. 2A and B) and the presence of several antigens typically expressed by MSCs and by their EVs (13), such as CD44, CD105, CD90 and α5-integrin (Fig. 2C). Regarding size distribution analyzed with NanoSight, LCD-EVs showed a size range of 180±73 nm not significantly different from the size distribution of unlabeled EVs (145±57 nm). DL-EVs showed a size of 250±89 nm with a second peak of larger size probably due to some aggregations occurring during the labeling procedure (Fig. 3A).

To evaluate the ability of labeled EVs to be incorporated by PTECs, 50 μg/ml of EVs labeled with the red dye, DiI, following the same procedure described above, were added to the cells. DL-EVs and LCD-EVs were equally incorporated within PTECs, as observed by confocal microscopy after 5 h of incubation (Fig. 3B).

### In vivo non-invasive OI visualization of EV biodistribution in AKI

**Figure 4:**
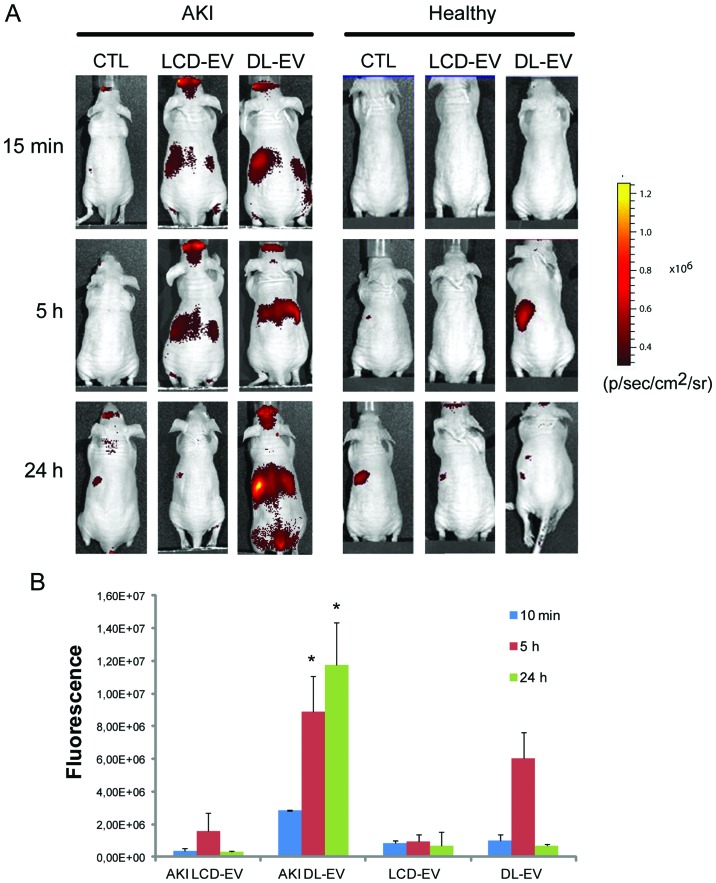
*In vivo* cell-derived extracellular vesicle (EV) biodistribution in kidney region by optical imaging (OI). (A) Representative OI images, acquired in the posterior position following the induction of acute kidney injury (AKI) in mice and in healthy mice treated intravenously with 200 μg of LCD-EVs or DL-EVs or with an equal volume of phosphate-buffered saline (PBS) (CTL). (B) Quantification of fluorescence intensity in regions-of-interest (ROI) draw free hand in the region of kidneys, expressed as the average radiance ± standard deviation (SD). Sixteen AKI mice were treated with LCD-EVs, 11 AKI mice were treated with DL-EVs; healthy mice received the same amount of LCD- and DL-EVs (n=12 for LCD-EVs and n=6 for DL-EVs). ANOVA with Newman*-*Keuls multi-comparison test was performed. ^*^p<0.01 AKI DL-EV vs. all the other groups. LCD-EV, labeled EVs produced by donor cells; DL-EV, directly labeled EVs.

**Figure 5:**
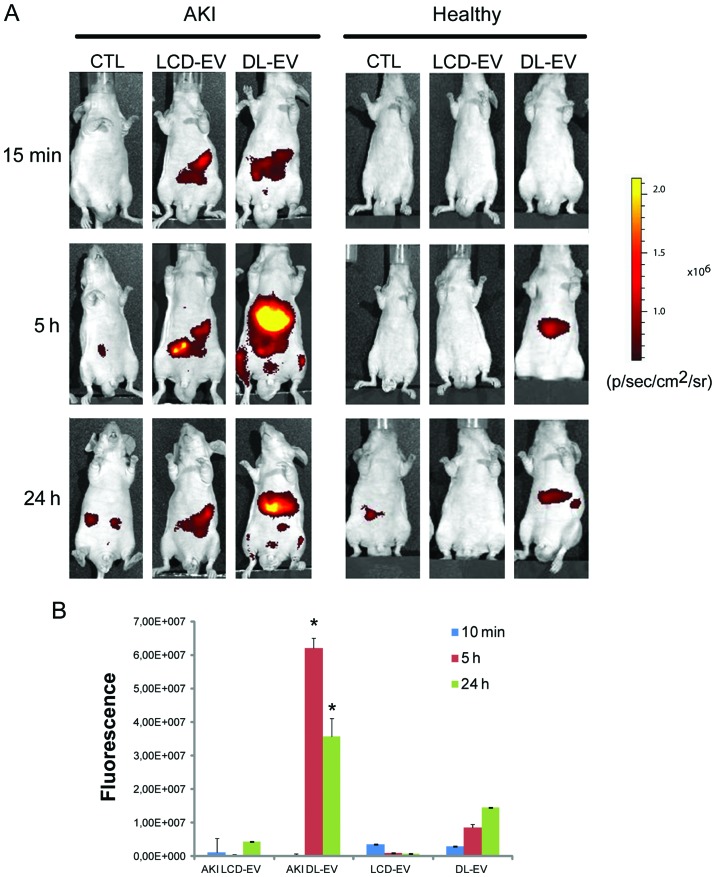
*In vivo* cell-derived extracellular vesicle (EV) bio-distribution in abdominal area by optical imaging (OI). (A) Representative OI images, acquired the supine position following the induction of acute kidney injury (AKI) in mice and in healthy mice treated intravenously with 200 μg of LCD-EVs or DL-EVs or with an equal volume of phosphate-buffered saline (PBS) (CTL). (B) Quantification of fluorescence intensity in regions-of-interest (ROI) draw free hand in the abdominal area, expressed as the average radiance ± standard deviation (SD). Sixteen AKI mice were treated with LCD-EVs, 11 AKI mice were treated with DL-EVs; healthy mice received the same amount of LCD- and of DL-EVs (n=12 for LCD-EVs and n=6 for DL-EVs). ANOVA with Newman*-*Keuls multi-comparison test was performed. ^*^p<0.01 AKI DL-EV vs. all the other groups. LCD-EV, labeled EVs produced by donor cells; DL-EV, directly labeled EVs.

The ability of labeled EVs to be visualized by OI on the whole body of live mice was assessed using an IVIS 200 system in a model of AKI induced by an intramuscular glycerol injection, as previously described (13). Three days after the glycerol injection, blood urea nitrogen (131±16 mg/dl) and creatinine (0.9±0.2 mg/dl) levels were significantly increased compared with the healthy controls (28±10 and 0.2±0.1 mg/dl, respectively) and were associated with diffuse tubular epithelial injury, characterized by tubular hyaline casts, vacuolization and widespread necrosis of the proximal and distal tubular epithelium, loss of brush border and denudation of the basal membrane (data not shown). The 200 μg of DL-EVs or LCD-EVs was inoculated i.v. 3 days following the induction of AKI, when functional and morphological damage had reached its peak (13). Healthy mice were treated with the same amount of EVs in order to evaluate whether the accumulation of EVs was specific for the site of injury. Fig. 4 shows a fluorescent signal in the region of kidneys of AKI mice treated with LCD-EVs and DL-EVs and analyzed posterior after 15 min and 5 h. However, the intensity of fluorescence in the DL-EV-treated mice with AKI was still present at 24 h after the i.v. injection and was significantly higher than the fluorescence signal generated by the kidneys of mice with AKI injected with LCD-EVs. In the healthy mice, we observed a fluorescent signal only in the mice treated with DL-EVs after 5 h in the left dorsal region that may correspond to spleen accumulation; however, the signal decreased rapidly and 24 h after the i.v. injection was not detectable (Fig. 4). Analyzing the signal in the abdominal area, a higher fluorescence intensity was observed in the AKI groups compared with the healthy groups. Nevertheless, the DL-EV-treated mice with AKI displayed a major increase in fluorescence intensity in comparison with the LCD-EV-treated AKI group, possibly due to the accumulation of EVs in the liver and spleen (Fig. 5).

### Ex vivo OI analysis of dissected organs

**Figure 6:**
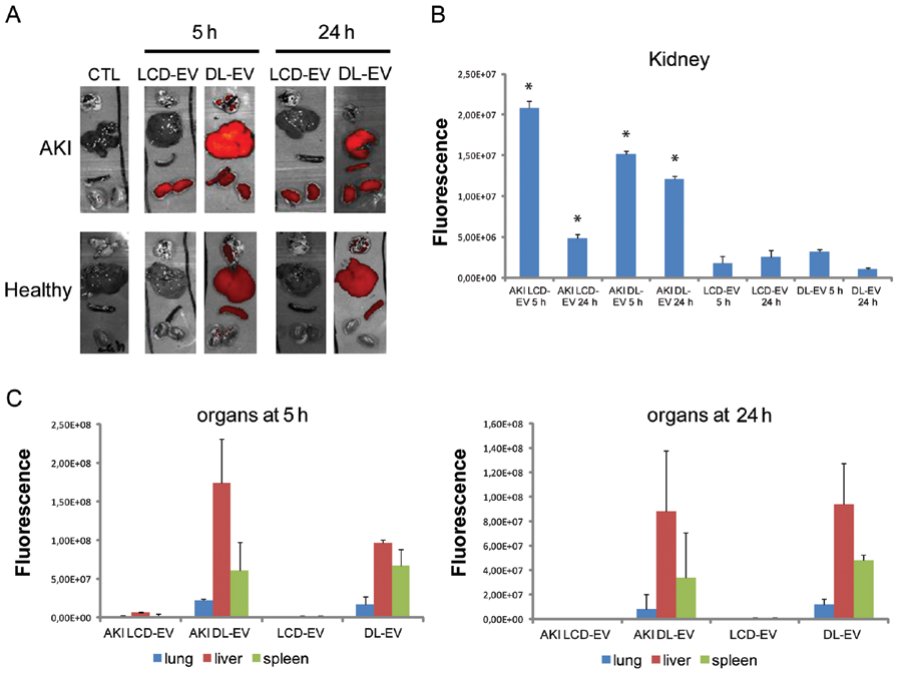
*Ex vivo* optical imaging (OI) analysis of dissected organs. (A) Representative OI images of dissected organs of mice with acute kidney injury (AKI) and healthy mice treated with LCD- and DL-EVs sacrificed at 5 and 24 h after injection. (B) Fluorescence quantification of kidneys, expressed as the average radiance ± standard deviation (SD). ANOVA with Newman*-*Keuls multi-comparison test was performed. ^*^p<0.05 AKI EVs vs. healthy EVs. (C) Fluorescence quantification, expressed as the average radiance ± SD, of lungs, liver and spleen. AKI mice treated with LCD-EVs sacrificed at 5 h (n=9) and at 24 h (n=7); AKI mice treated with DL-EVs sacrificed at 5 h (n=6) and at 24 h (n=5); healthy mice treated with LCD-EVs sacrificed at 5 h (n=6) and at 24 h (n=6); healthy mice treated with DL-EVs sacrificed at 5 h (n=3) and at 24 h (n=3). LCD-EV, labeled EVs produced by donor cells; DL-EV, directly labeled EVs.

For each experimental group, the mice were sacrificed at 5 and 24 h after the EV injection and the fluorescent signal from freshly dissected tissues was quantified immediately by OI. The fluorescence intensity of the kidneys of mice with AKI treated with LCD-EVs and DL-EVs was significantly higher after 5 h compared with the kidneys of mice with AKI treated with PBS [AKI CTL (control)], as shown in Fig 6. Nevertheless, the increase in fluorescence was significantly maintained for 24 h following treatment in the DL-EV group, whereas it decreased in the LCD-EV group (Fig. 6A). No signal was detected in the kidneys of healthy mice treated with LCD-EVs and DL-EVs, suggesting a specific accumulation of EVs at the site of injury.

The fluorescence signal of DL-EVs was also detected in the spleen and particularly in the liver with high variability. The fluorescence signal of DL-EVs was of low intensity in the lungs of both the AKI and healthy groups. LCD-EVs were detectable only in the injured kidneys.

**Figure 7:**
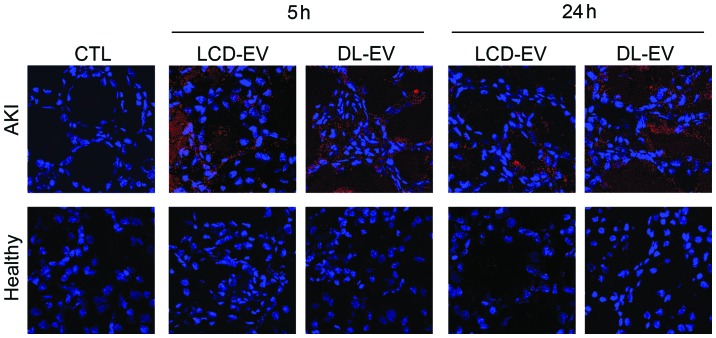
Confocal microscopy of fluorescent cell-derived extracellular vesicles (EVs) in kidneys. Representative micrographs of kidney sections of mice with acute kidney injury (AKI) and healthy mice sacrificed at 5 and 24 h after EV injection (red). Nuclei were counterstained with Hoechst dye. Two kidney specimens were analyzed for each experimental point. Original magnification, ×630. LCD-EV, labeled EVs produced by donor cells; DL-EV, directly labeled EVs.

The presence of LCD-EVs and of DL-EVs within injured kidneys was confirmed by confocal analysis using the appropriate wavelength (Fig. 7). In the kidney sections derived from healthy mice, the presence of fluorescent EVs was almost absent.

## Discussion

The results of the present study demonstrate that it is possible to analyze the biodistribution of EVs either by direct labeling or by the production of labeled EVs from MSCs. In particular, labeled MSC-derived EVs were found to localize within the injured kidneys.

The imaging of EVs *in vivo* may contribute to understanding the regenerative potential of EVs released from stem cells. Different approaches to visualize EVs have been proposed, exploiting fluorescent protein-based imaging, such as green fluorescent protein GFP (34), red fluorescent protein (RFP) (35), or the enzymatic activity of the luciferase enzyme which is secreted within exosomes (36). Nevertheless, the use of fluorescent proteins limits the visualization only to the EVs that possess the candidate proteins. Therefore, this technique cannot be applied to all type of vesicles. Since extracellular vesicles are a broad group, differing in content, size and surface markers (37,38) we used a technique that allows the labeling of EV lipid membranes.

The use of small-molecule fluorophores and, in particular, NIR molecules, is a powerful tool to track EVs for non-invasive visualization. These dyes present strong and stable fluorescence in the EV membrane (28). NIR molecules exert a high tissue penetration in concomitance with a low background signal. These dyes have been employed for *ex vivo* EV detection (28). Since the possibility to trace these dyes *in vivo* has been shown for labeled cells and antibodies (33,39), in this study, we assessed the possibility to label EVs with DiD for EV tracking.

Previous publications addressing the biodistribution of EVs, have used the *ex vivo* detection of the dyes in dissected organs (28). Takahashi *et al* (36), using the luciferase activity of EVs, described the possibility to monitor their biodistribution within 4 h.

In this study, we compared the efficiency and sensibility of two labeling methods to visualize EVs in living animals. DiD-labeled EVs were obtained by direct labeling after their production or from the supernatant of MSCs previously incubated with DiD. Labeled EVs were administered to a mouse model of AKI induced by a glycerol injection and compared with healthy controls, to observe their biodistribution. EVs derived from human MSCs have been shown to accelerate the recovery of AKI in different mouse models (13,19,20). In the present study, we found that labeled EVs accumulated specifically in the kidneys of mice with AKI compared with healthy controls. After 5 h, the EVs were detectable in whole body images and in dissected kidneys by OI using both types of labeling procedure. However, the DL-EVs showed a higher and brighter fluorescence compared with the LCD-EVs. In the whole body, the signal generated by the DL-EVs was maintained for 24 h after the injection, whereas the signal of the LCD-EVs was detectable only in the dissected kidneys. Moreover, the liver and spleen of the mice treated with DL-EVs possessed a fluorescence signal due to a non-specific accumulation of EVs in the excretory organs. Comparing the two methods, the LCD-EVs showed a greater specificity due to their detection only in injured tissue, but the intensity of the fluorescence was lower than that of the DL-EVs. It is known that MSCs are recruited at the site of injury by receptor-mediated interaction (16). MSC-derived EVs bear the same membrane receptors of MSCs; it is therefore possible that they may accumulate at the site of injury by exploiting the same mechanisms. In addition, the increased permeability in the injured kidney may account for the local accumulation of EVs in AKI.

In conclusion, both these labeling methods were found to be suitable for the *in vivo* detection of the renal localization of EVs. The localization of EVs in diseased, but not normal kidneys, may explain their beneficial effects on recovery following AKI.
